# Food matters: how the microbiome and gut–brain interaction might impact the development and course of anorexia nervosa

**DOI:** 10.1007/s00787-017-0945-7

**Published:** 2017-01-31

**Authors:** Beate Herpertz-Dahlmann, Jochen Seitz, John Baines

**Affiliations:** 1 Department of Child and Adolescent Psychiatry, Psychosomatics and Psychotherapy, University Clinics, Technical University RWTH, D-52074 Aachen, Germany; 2 Institute for Experimental Medicine, Christian-Albrechts-University of Kiel and Max Planck Institute for Evolutionary Biology, Plön, Germany

**Keywords:** Anorexia nervosa, Microbiome, Gut–brain interaction, Starvation, Autoimmune disease

## Abstract

Anorexia nervosa (AN) is one of the most common chronic illnesses in female adolescents and exhibits the highest mortality risk of all psychiatric disorders. Evidence for the effectiveness of psychotherapeutic or psychopharmacological interventions is weak. Mounting data indicate that the gut microbiome interacts with the central nervous system and the immune system by neuroendocrine, neurotransmitter, neurotrophic and neuroinflammatory afferent and efferent pathways. There is growing evidence that the gut microbiota influences weight regulation and psychopathology, such as anxiety and depression. This article reviews how the gut–brain interaction may impact the development and course of AN. A “leaky gut”, characterized by antigens traversing the intestinal wall, was demonstrated in an animal model of AN, and could underlie the low-grade inflammation and increased risk of autoimmune diseases found in AN. Moreover, starvation has a substantial impact on the gut microbiome, and diets used for re-nutrition based on animal products may support the growth of bacteria capable of triggering inflammation. As there is currently no empirically derived agreement on therapeutic re-nourishment in AN, this review discusses how consideration of gut–brain interactions may be important for treatment regarding the determination of target weight, rapidity of weight gain, refeeding methods and composition of the diet which might all be of importance to improve long-term outcome of one of the most chronic psychiatric disorders of adolescence.

## Introduction

The Hippocratic concept of positive health dates back to the fifth century B.C. and postulates that adequate nutrition is an important component of a long and healthy life [1]. Nutrition comprises macronutrients (carbohydrates, proteins and fat) and micronutrients (vitamins and minerals) to supply energy and enable an organism to grow and function properly. Although most people consider sufficient and balanced eating as a basic drive to maintain well-being, anorexia nervosa (AN) is characterized by insufficient food intake and poor diet, which lead to a significantly low body weight and severe danger to the individual’s health. Additionally, patients with AN suffer from severe weight phobia; in many but not all cases, there is a lack of recognition of the seriousness of the illness [2].

Some 30 years ago, the aetiology of AN was explained by a psychosomatic family model [3, 4]. Herein, nutritional rehabilitation and weight gain were not considered to be of primary importance to the healing process. Today, it is well-known that a higher body mass index (BMI) at the end of treatment correlates with a better overall outcome [5] and that a normalization of body weight is necessary to prevent severe somatic sequelae such as osteoporosis and infertility [6, 7]. Moreover, it is well-established that weight gain is a potent agent against comorbid mental disorders of AN, especially depression [8, 9].

Most mental disorders emerge by an interaction between a person's biological disposition and environmental influences, i.e. by an interaction of “nature” and “nurture”. In AN, the latter is to be taken literally. In animals and healthy individuals, eating is both a motivating and appetitive behaviour that is highly reinforced and rewarded by certain neurocircuits in the brain (for a review, see [10]). However, this “core eating network” [10] seems to be severely affected in patients with eating disorders, especially AN. These individuals seem to process food cues differently in comparison to healthy eaters, exhibit decreased anticipation of food taste and often show strong regulatory responses enabling control and restraint of food intake [10]. However, it remains unclear whether these changes are primary in origin, and thus a cause of the eating disorder, or whether they appear during the illness as secondary effects.

There is emerging evidence of important links between the gut microbiome and the CNS, which might be depicted as “a gut feeling for the brain” [11] (for a review, see [12]). Humans sustain a symbiotic relationship with the microbiome in their gut, which consists of approximately 10^14^ cells; thus there are ten times more bacteria than cells in the average human body [13]. The highest bacterial concentrations are found in the colon with approximately 10^12^ per gram [14]. We supply our microbiota with food, and they compensate us with important health benefits in relation to digestion, growth and defence against pathogens [11, 15].

We have recently learned that malnutrition and long-term dieting have a substantial and reproducible effect on the gut microbiome and its impact on the brain, which is likely related to the development of psychopathology and psychiatric disorders [15]. There is growing evidence that the gut microbiome also plays an important role in the development and persistence of eating disorders, especially AN [16].

Among all age groups, less than 50% of patients with AN fully recover [17], and the mortality risk is the highest of all psychiatric disorders [18]. Medication has a very limited, if any role in the treatment of AN, and psychotherapeutic interventions are only moderately effective (for a review, see [19]). To make progress in treatment, a more “brain-directed therapy” [20] is likely necessary to overcome this often disabling disease.

Thus, the aim of this article is to present recent findings on the gut–brain interaction that may be of relevance to the pathophysiology of eating disorders, and for AN in particular. In part because this research is still at an early stage, several results are contradictory, and most findings are based on animal models that may not be easily transferred to humans. While we do not fully review all substantial aspects of gut–brain interaction for weight regulation or psychiatric disorders, our assessment aims to focus on the influences of malnutrition, the process of refeeding and their presumable impact on the course of AN. “Nutritional medicine” and microbiota-modulating strategies may be promising determinants of the healing process and outcome of AN.

## Communication pathways between the gut microbiome and the brain

The gut is the most heavily bacterially colonized area of the human body, with greater bacterial numbers in the colon than in the upper intestine (see above), in addition to differences in composition between the bacteria in the gut lumen and those near the mucus layer [21]. One of the most important “tasks” of the gut microbiome is contributing to the protection of the intestinal barrier, which must prevent the passage of pathogenic microorganisms and toxic substances into systemic circulation. Certain periods of life seem to involve particular vulnerability to changes in gut permeability, e.g. early life and old age [22]. There are several pathways by which changes in the intestinal barrier might impact brain homeostasis, particularly neuroendocrinological alterations, neurotransmission, neurogenesis and neuroinflammation, either by direct passage to the brain or by the vagus nerve, which acts as the main pathway from the lumen of the gut to the nucleus solitarius in the medulla oblongata [11].

### Neuroendocrinological pathways—Hypothalamic–Pituitary–Adrenal (HPA)-axis

In animal models and human studies, the experience of stress is linked to an increase in the permeability of the intestinal barrier, which might be mediated by—among other factors—hypothalamic hormones, especially CRH [22]. Acute stress increases gastrointestinal and blood–brain barrier permeability through the activation of mast cells, which express high-affinity receptors for CRH [15]. Conversely, in germ-free (GF) rodents, higher levels of ACTH and CRH are observed after exposure to stress compared to conventionally colonized animals, suggesting that the gut microbiome contributes to the downregulation of the HPA-axis. Notably, this effect appears dependent on specific bacterial taxa, as only certain species such as *Lactobacillus salivarius* were able to attenuate the stress response [11].

In humans, even relatively small acute stress situations such as public speaking are followed by an increase in intestinal permeability, although only in those who also respond with elevated cortisol levels [23].

AN is associated with elevated serum, urinary and salivary cortisol levels in the acute state, as well as with a lack of cortisol suppression on overnight dexamethasone and dexamethasone suppression-CRH stimulation testing [24, 25]. Thus, it may be hypothesized that AN-specific aberration of the HPA-axis could contribute to the dysfunction of the intestinal barrier that is observed in an animal model of AN (see below, [26]).

### Neurotransmission

It is increasingly recognized that gut microorganisms have a notable effect on the development and regulation of peripheral and central serotonergic function, especially in the hippocampus. Yano et al. [27] propose a model in which products of microbial fermentation, e.g. short-chain fatty acids (SCFA, see below) and bile acids (see below), directly act on enterochromaffin cells, and thus promote the release of serotonin, which influences gastrointestinal mobility and platelet function. GF mice display depressed levels of serotonin in plasma compared to conventionally raised mice [28, 29]. However, they display less anxiety- and depression-like behaviour than naturally raised mice [30, 31]. Anxiety and depression are closely linked to disturbed serotonin metabolism [32]. In contrast to the findings by Wikoff et al. [28] in plasma extracts of GF mice, significantly increased hippocampal levels of 5-hydroxytryptamine (5-HT) and 5-hydroxyindoleacetic acid (5-HIAA), the main metabolites of serotonin, were found in developing GF mice compared to naturally raised mice. A sustained absence of microorganisms in these animals was observed to induce a 1.3-fold increase in 5-HT, which compares to that induced by antidepressants such as SSRIs (for a review, see Clarke [33]). This increase appears to be sex-specific as it was only found in developing male GF mice. The reasons for this sex difference are not well explored, but are probably linked to the menstrual cycle and the influence of oestrogen on the serotonergic system. Notably, elevated levels of 5-HT are maintained when these mice are colonized with a normal microbiota later in life, which also demonstrates that the early life period plays an important role in configuring the gut–brain interaction [33].

A recent study in patients with major depression demonstrated that patients differed from normal controls in either a predominance of some potentially harmful gut bacteria or a reduction of beneficial bacterial groups [34]. Further, a large evaluation of medical record-based data in the UK showed that repeated use of antibiotics was associated with a significant increase in depressive and anxious symptoms [35].

It is suspected that the serotonergic system is severely altered in AN. During the active state of the illness, patients have a significant reduction in CSF-5-HIAA in comparison to healthy controls; in long-term weight-restored patients, CSF-levels of 5-HIAA are elevated, which is generally related to disorders of anxiety and obsession [36]. A dysfunction of the serotonergic system involving 5-HT-receptors and 5-HT-transporters in AN was also confirmed by brain-imaging studies (for a review, see [37]). However, medications targeting serotonergic dysfunction, such as SSRIs, are not proven effective in AN (for a review, see [38]).

### Neurogenesis

Brain-derived neurotrophic factor (BDNF) is a nerve growth factor known to influence neuronal development, increase synapse plasticity especially in the hippocampus, and confer protection against stress-induced damage [39]. BDNF function is linked to mood, stress tolerance and cognitive function such as memory. Recent animal research suggests that the gut microbiome may influence the expression of BDNF in the brain, although the mechanism is not yet clear [11]. Prolonged administration of antibiotics results in a reduction [40], while pre- or probiotics (see below) seem to enhance hippocampal BDNF levels [41, 42]. In the absence of gut bacteria, the expression of BDNF in male mice is lowered in comparison to conventionally colonized mice, while it is increased by prebiotic feeding [43].

We are unaware of any studies on the association between the microbiome and BDNF in humans.

In AN, BDNF levels are reduced in acutely ill patients [44], but increase with short-term weight gain [45]. Thus, it could be hypothesized that diet-associated changes in the microbiome contribute to different stage-related levels of BDNF in the brains of AN patients, which might also influence cognitive functioning (e.g. psychomotor speed) [45]. Möhle et al. [46] directly showed that neurogenesis in the hippocampus was reduced in mice after antibiotic treatment, which resulted in impaired memory functions, whereas probiotic intake completely restored neurogenesis and memory functions. However, it remains to be demonstrated whether similar phenomena occur in humans.

### Neuroinflammation

The intestinal barrier normally prevents pathogenic microorganisms from spreading into systemic circulation. However, the so-called “leaky gut” (increased intestinal permeability) might facilitate the transfer of potentially pathogenic members of the microbiota (i.e. “pathobionts”), metabolites, toxins or lipopolysaccharides from the gut lumen to the lamina propria and on to the mesenteric lymph nodes, from which they may reach systemic circulation, especially in the case of an aberrant immune response [12, 15, 47]. This phenomenon was observed both in animal models for depression [48] and in depressed humans [47]. Once in circulation, the bacteria and other gut luminal contents stimulate the output of pro-inflammatory peripheral and central cytokines, the latter influencing neuronal function. Enhanced levels of IgA- and IgM antibodies against bacterial lipopolysaccharide, the structural component of the external membrane of Gram-negative bacteria, are observed in depressed patients, especially in those with chronic disorders [49]. However, it is not yet clear what comes first; increased gut permeability could induce mucosal inflammation followed by systemic inflammation; conversely, systemic inflammation could disturb intestinal barrier function, thus leading to an increased bacterial translocation and fuelling of systemic inflammation [12].

An increased risk for autoimmune disorders is observed in AN, especially for those involving the gastrointestinal tract (see below, [50]).

### Short-Chain Fatty Acids (SCFAs) with neuroactive qualities

Short-chain fatty acids mainly represent the product of fermentation of partially digestible and non-digestible carbohydrates, namely dietary fibre, by the microbiome [51]. The most important SCFAs are acetic acid, propionic acid and butyric acid, which can act as “signalling molecules” and affect the physiology of the host organism, such as influencing the ph-level of the colon, controlling the gut transit time, metabolizing glucose and modifying appetite and energy homeostasis [11, 52–54]. SCFAs are also able to cross the brain–blood barrier and impact neural circuits [55]. They are involved in regulating the expression of neuropeptides, such as PYY and ghrelin [56] and are associated with antidepressant effects in animal models via higher concentrations of BDNF.

## Starvation and the gut microbiome

Mouse models provide evidence that the transfer of microbiota from genetically or nutritionally induced obese mice to GF mice can lead to obesity and associated metabolic disturbances in the host animals [57, 58]. In contrast, kwashiorkor is a severe form of acute malnutrition often observed in developping countries that is probably worsened by a protein-deficient diet. It was recently shown that the consequences of this type of malnutrition are affected by the gut microbiome and vice versa [59]. Frozen bacterial species from children with kwashiorkor could be transplanted into GF mice; the combination of the transplanted kwashiorkor species with the African diet produced significant weight loss in these mice associated with severe metabolic disturbances. Although symptoms of kwashiorkor could be alleviated by better nutrition, symptoms returned when the African diet was re-implemented.

Starvation-induced changes in the gut microbiome were also found in an acute and chronic starvation animal model using activity-based anorexia (ABA), which is the most widely utilized rodent model of AN simulating weight loss by food restriction and hyperactivity with the help of access to a running wheel (e.g. [60]). After the ABA mice lost a substantial amount of weight (approximately 20%), a histological investigation of the colon revealed decreased thickness of the muscularis layer and significantly increased permeability of the colon [26]. These alterations observed in an animal model for AN suggest that intestinal barrier dysfunction provoked by starvation might also contribute to the pathophysiology of AN. In contrast, in an earlier study of AN patients by Monteleone et al. [61], a decrease in intestinal permeability was found. However, the authors of this earlier study used the administration of an oral sugar solution of Lactulose/Mannitol excreted in the urine for their testing, which mainly examines the permeability of the small intestine [62]. In the study of ABA mice by Jesus et al. (2014), the permeability of the colon assessed by histological analysis was increased. Moreover, the amount of the oral dose of the sugar absorption test excreted in the urine does not only depend on the permeability of the intestinal mucosa, but also on other factors, such as gastric emptying, intestinal transit time and renal clearance [62], all of which may be disturbed in AN.

In addition, several studies demonstrated an increase in intestinal permeability during exercise, which is found in the majority of patients with AN, and also represents a basic mechanism in the ABA model [63].

A disturbed gut barrier function was also found in other disorders associated with malnourishment and in volunteered fasting subjects [64, 65].

## Previous studies in AN

Until now, our knowledge of the relevance of the gut microbiome for the symptomatology of AN is scarce. Very few studies investigated the composition of the intestinal microbiome during the acute and recovered states of AN. A single culture-based case study of an AN patient at admission identified 11 previously unknown bacterial species [66], whereas a study of 25 patients with AN found a reduced number of total bacteria and obligate anaerobes [67]. Armougom et al. [68] compared the gut microbiome in obese individuals, healthy controls (HC) and AN patients and found an increased amount of *Methanobrevibacter smithii* in AN [68].

The first longitudinal results from AN were presented by Kleiman et al. [69], who analysed the faecal microbiome in a small number of female, mostly adult AN patients collected at admission (*n* = 16) and discharge after weight gain (*n* = 10) and compared it to HC samples. Weight loss at admission was of medium severity. At discharge patients (who could be reassessed twice) were still at a rather low weight. In comparison to HC, patients with AN displayed significantly reduced alpha diversity (describing the within-sample diversity) in the state of a low BMI. After weight gain, there was an increase in alpha diversity, although it remained lower than in HC [69]. Notably, differences in overall diversity and the abundances of individual bacterial groups were significantly associated with eating disorder psychopathology and depression scores, a finding consistent with the results of other studies showing associations between the gut microbiome and behaviour (e.g. [34]).

In a very recent German study from the University of Tuebingen [70], the results of Kleiman et al. [69] were for the most part confirmed in their much larger sample of 55 individuals, which were predominantly adults with a mean BMI of approximately 15 kg/m^2^. Although alpha diversity was lower in AN patients than in HC at admission, the difference was not statistically significant. Furthermore, as already observed in the study by Kleiman et al. [69], there was again a significant increase in alpha diversity in AN patients after weight gain. Nevertheless, AN patients’ microbiome after weight gain still resembled that of patients at the beginning of treatment more than that of HC in terms of beta diversity (inter-individual variability). However, BMI was still very low (mean BMI 17.7) at the second assessment point, so that the effect of a more normalized body weight could not be investigated. Importantly, the abundances of individual taxonomic groups differed significantly between AN patients and HC, which included an increase in mucin-degraders (e.g. Verrucomicrobia and *Bifidobacteria*) and a decrease in butyrate-producers (e.g. *Roseburia* spp.) in AN patients. Mucin-degraders feed on mucus covering the intestinal wall and potentially contribute to the “leaky gut” identified in the ABA animal model of AN (39) mentioned above. Moreover, the authors analysed the production of short-chain fatty acids (see above) before and after weight gain. The concentration of the sub-group of branched-chain fatty acids, a product of protein fermentation, was increased in AN, although total SCFA production did not differ. They further note that branched-chain fatty acids can have negative effects on host physiology, e.g. by impacting PYY-releasing cells, and even psychopathology, e.g. by increasing depressive symptoms [70, 71].

**Fig. 1:**
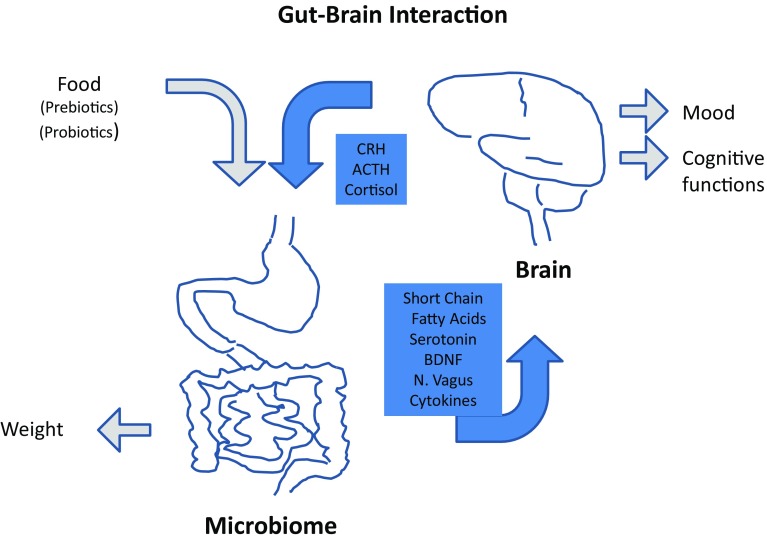
Interaction between the gut microbiome and the brain. While food is one of the main influencing factors of the gut microbiome, the microbiome in turn is an important influencing factor of body weight. Multiple interactions link the microbiome with the brain: Gut originating fatty acids (SCFA´s), serotonin, brain-dervied neurotrophic growth facotr (BDNF), nervus vagus stimulation and inflammatory cytokines appear to impact the brain via various pathways and infuence mood and memory function. In turn, brain originating corticotropin-releasing hormone (CRH), adrenocorticotropic hormone (ACTH) and cortisol influence the gut and its microbiome. In AN, a “leaky gut” seems to increase transfer of microbiotal antigens into the systemic circulation, further increasing inflammation and influencing interactions with the brain, potentially also affecting mood and memory function

As the peak onset of AN is in adolescence [72], the effect of young age and pubertal changes on the microbiome should be explored. An influence of age on the microbiome was just below the significance threshold in the study by Mack et al. [70]. However, only 11 subjects were below the age of 18, including five below the age of 16, and profound hormonal changes, which might influence the development of the microbiome [73], emerge between the age of 12 and 15 years. Thus, it might be important to build on the results by Mack et al. [70] and also study younger adolescents (Fig. 1).

## Gut permeability, inflammation and anorexia nervosa

A relationship between immune-mediated disorders and the gut microbiome is the subject of recent discussion. The microbiome stimulates the release of cytokines and other inflammatory mediators [11]. A “leaky gut”, which can be provoked by starvation and increases in mucin-degrading bacteria, is characterized by antigens traversing the intestinal wall, infiltrating systemic circulation, and thus contributing to a chronic low-grade inflammation presumed to be present in AN. Interestingly, autoantibodies against neuropeptides associated with appetite and stress regulation have been identified in AN [74, 75]. Moreover, a Finnish study conducted between 1995 and 2010 with a large eating-disorder cohort (*n* = 2342) demonstrated an elevated risk for autoimmune diseases in these patients [50]. According to their dataset assessing 30 autoimmune diseases, the most frequent were of endocrinological and gastroenterological origin. The lifetime risk (OR) of patients with an eating disorder (all diagnoses) to also suffer from an endocrinological disease, mostly diabetes mellitus type 1, was more than twice as high (OR 2.4) as for HC, while the risk of suffering from a gastroenterological disease was nearly twice as high (OR 1.8). The highest rates were found for an association with Crohn's disease (OR 3.09). In other previous studies, pro-inflammatory cytokines were shown to be increased in acute AN, although they mostly returned to normal after nutritional rehabilitation [76] (meta-analysis by Solmi et al. [77]). However, they may play a pivotal role in primarily chronic courses. In a recent case report of a young woman with co-existing Crohn’s disease and AN, the latter was much improved by the prescription of anti-TNF-alpha treatment [78].

Similar to a putative role in irritable bowel syndrome, increased gut permeability might also be associated with gastrointestinal complaints frequently occurring in acute and chronic AN [79, 80]. However, in the study by Mack et al. [70], gastrointestinal symptoms improved, but did not completely alleviate with weight gain, although microbial richness increased.

## Implications for treatment

As discussed above, there is substantial evidence for alterations of the gut microbiome in eating disorders, especially AN. These new insights might lend support for new therapeutic targets in this often chronic disorder, such as defining the right target weight, modifications of the refeeding process and the composition of the diet, non-bacterial dietary supplements and possibly the future use of pre- or probiotics (see below).

### Target weight

Although restoration of a healthy body weight is an important goal in the treatment of AN, there is no empirically derived agreement on the definition of an appropriate target weight (for a review, see [81]). However, with regard to body mass index (BMI), the gut microbiome plays an important role. Million et al. [82] analysed the association between the gut microbiome and body weight and found that the proportion of certain bacteria was significantly correlated with BMI. They compared four bacterial species in the following four weight classes: obese, overweight, lean and anorectic individuals. The abundance of *Lactobacillus* species was positively correlated with BMI, while *Bifidobacterium animalis*, *Methanobrevibacter smithii* and *Escherichia coli* were negatively correlated with BMI. Thus, given the differences observed in previous studies of AN (i.e. an increase in mucin producers and decrease in SFA-producers, [70]), it might be possible that the composition of the gut microbiome could be used to help determine individualized target weights for patients (e.g. in the context of a restoration of bacterial community structure).

### Refeeding and composition of diet

Refeeding practices in AN are mostly based on mainstream clinical or expert opinions—there is little empirical evidence for a healthy weight regimen in affected individuals. While European clinicians usually start patients on a low-calorie diet to avoid the so-called refeeding syndrome, American practitioners are less cautious and suggest a much higher calorie supply even at the beginning of refeeding (for a review, see [81]).

According to the findings of the Kaye group [83], patients with the restricting type of AN need a higher quantity of calories to gain the same amount of weight as patients with the bulimic type. While this difference is mostly ascribed to higher energy expenditure in patients with the restrictive type compared to those with the bulimic type, additional involvement of the gut microbiome is conceivable. Indeed, Mack et al. [70] confirmed significant differences in microbial community composition according to AN subtype. Binge/purge patients often have a higher premorbid body weight, which might be associated with a different composition of the gut microbiome. The latter is a key determinant of the transformation of bile acids to efficiently take up lipids, carbohydrates and fatty acids [84]. The microbiome of both obese mice and humans has an increased capacity to harvest energy from nourishment [57, 85–87]. Faecal samples transplanted to GF mice from obese humans led to increased body and fat mass accompanied with obesity-associated metabolic dysfunctions [58]. However, normalization of body weight and obesity-related metabolic perturbations was achievable through co-housing with lean mice (and hence, exposure to their gut microbes through coprophagy) combined with a diet rich in vegetables and fruit but low in saturated fats. Thus, one might speculate that augmenting gut microbial composition in combination with certain diets may have potential to improve weight restoration in AN.

The composition of the human microbiome has been linked to long-term dietary patterns, such as diets in Western (animal protein, sugar and fat) and Non-Western populations (plant-derived carbohydrates) [80, 88]. However, the human gut microbiome seems to respond rapidly to rigorous short-term macronutrient changes, whereby individual diet-driven changes can occur within 3–4 days, but reverse in a similar time frame [89]. In an important study by David et al. [90], the authors compared the effects of a shift in macronutrient intake from usual eating habits to either an animal-based diet or a plant-derived diet. Changes in the composition of the gut microbiome occurred only one day after the new diet had reached the distal gut, but exclusively in the group on the animal-based diet, and the gut microbiome returned to its usual composition two days after this diet ended. Although there was no difference in alpha diversity between the two groups, beta diversity (difference in community structure between baseline and diet) significantly changed in the animal-based food group. Notably, in contrast to the participants on the vegetarian diet, those on the animal-based diet showed significant weight loss, although caloric intake was similar in both groups. One of the most abundant phyla in the microbiota of the animal-based diet group was a bacterium with a high bile resistance (*Bilophila wadsworthia*), which is in accordance with our knowledge that a diet rich in fat results in higher bile acid production. In mice, growth of *B. wadsworthia* is stimulated by ingesting saturated fats from milk; it is important that the expansion rate of *B. wadsworthia* is also associated with the development of inflammatory bowel disease in these mouse models [90, 91]. Thus, we may hypothesize that certain diets—also in humans—will contribute to the pathogenesis of bowel inflammation.

Why are these findings important for the treatment of AN? Before admission, anorexia nervosa patients often maintain a vegetarian diet low in fat and high in fibre [92]. After hospitalization, diet is often quickly changed to a high-calorie diet rich in carbohydrates and fat for nutritional rehabilitation. Moreover, in cases of very severe AN, patients are sometimes tube-fed or are given oral liquid supplements. Most oral liquid supplements, especially those suitable for tube feeding, are based on cow’s milk, e.g. an animal-based food product. Thus, within a very short time, we may strongly affect the gut microbiome of our patients without being aware of the consequences, such as the possible growth of inflammation-inducing bacteria. Although two studies investigated the impact of weight gain on the gut microbiota (see above, [69, 70]), none so far has explored the effect of the diet itself.

### Pre- and probiotics

Gibson and Roberfroid [93] defined a prebiotic as a “nondigestible food ingredient that beneficially affects the host by selectively stimulating the growth and/or activity of one or a limited number of bacteria in the gut”. Currently, the selectivity criterion is left out, and a prebiotic is defined as a “substance that induces the growth of microorganisms that contribute to the well-being of their host” (Wikipedia, assessed 7/7/2016 [94]; for a review, see [95]). The most well-known prebiotics to impact the gut–brain axis are fructans (such as inulins and oligofructose) and glucans (such as galacto-oligosaccharides) [11]. The latter have been demonstrated to improve intestinal barrier function in rodents [96]. More recently, substances such as pectins and milk oligosaccharides are also included among prebiotics. It is hypothesized that metabolic function of the organism can be improved and low-grade inflammation reduced by altering the gut microbiome with the help of prebiotics [87].

Other studies suggest that prebiotics may be effective in the treatment of depressive or anxious states and cognitive function. Glucans and polydextrose were shown to reduce anxiety-related behaviour in rats [97] and to attenuate stress response in healthy adult probands [98].

A probiotic is defined as living component of the microbiota administered to humans or animals that are associated with health benefits for the host. In several studies, a reduction of intestinal permeability was reported, e.g. in pre-term infants, in children with atopic dermatitis and irritable bowel syndrome after application of probiotics (for a review, see [12]). Möhle et al. [46] showed that after severely reducing the gut microbiome with several antibiotics, neuropsychological deficits, such as learning impairments and reduced neurogenesis in the hippocampus, could be induced in mice. These impairments could be reversed by orally administering an over-the-counter mix of probiotic bacteria. Although an effect of probiotics on depressive- and anxiety-like behaviour in mice has been observed [99], reports involving larger human samples are still lacking. Mazurak et al. [100] analysed the evidence of probiotic use in irritable bowel syndrome and criticised that studies suffer from heterogeneity in sample sizes, duration of treatment and concentration of probiotics. In a systematic review by Kristensen [101] including seven studies between 2013 and 2015, no significant effect of probiotics on faecal microbiota composition in healthy adults was found when compared to placebo. Moreover, there are no studies on the benefit of pre- or probiotics in AN.

It is beyond the scope of this article to discuss the benefit of pre- or probiotics in more detail. Sheridan and coauthors [102] assert that the type of malnutrition should be strictly defined and the target of the intervention should be specified before administering pre- or probiotics to undernourished patients. In addition, it should be kept in mind that there are differences in the type of malnutrition caused by famine, old age or somatic disorders in comparison to AN patients, who commonly ingest “healthy low caloric food” such as a vegetarian diet, which will most likely also result in different effects on the microbiome.

## Conclusions

There is growing evidence that the gut microbiome plays a notable role in the emergence and development of somatic and psychological symptoms in AN. The consideration of microbe–gut–brain interactions as risk factors for the onset and perpetuation of AN and as a target for therapeutic interventions, will likely prove to be a significant shift in our scientific concept of the aetiology and treatment of eating disorders. Until now, there have been very few studies on the role of the intestinal microbiome in AN and other eating disorders. Accordingly, we are not aware of the consequences of a diet rapidly changing in caloric content and composition. Future research should include continuous monitoring of the microbiome during nutritional rehabilitation, weight gain and hormonal restoration far beyond previous assessments at only the beginning and end of treatment. Our knowledge on the effect of rapid weight gain, changes in macronutrients and target weight level on the microbiome and consequent inflammation mechanisms is scant. Moreover, our insight into gut–brain interaction, especially in adolescent AN, is very limited. However, the exploration of gut–brain communication and its alteration, especially in the early stages of the disease in adolescence, may be particularly important for the prognosis of this often long lasting and disabling disorder.
